# A European Research Agenda for Somatic Symptom Disorders, Bodily Distress Disorders, and Functional Disorders: Results of an Estimate-Talk-Estimate Delphi Expert Study

**DOI:** 10.3389/fpsyt.2018.00151

**Published:** 2018-05-14

**Authors:** Christina M. van der Feltz-Cornelis, Iman Elfeddali, Ursula Werneke, Ulrik F. Malt, Omer Van den Bergh, Rainer Schaefert, Willem J. Kop, Antonio Lobo, Michael Sharpe, Wolfgang Söllner, Bernd Löwe

**Affiliations:** ^1^Clinical Centre of Excellence for Body, Mind, and Health, GGz Breburg, Tilburg, Netherlands; ^2^Tranzo Department, Tilburg University, Tilburg, Netherlands; ^3^Sunderby Research Unit, Division of Psychiatry, Department of Clinical Sciences, Umeå University, Umeå, Sweden; ^4^Institute of Clinical Medicine, University of Oslo, Oslo, Norway; ^5^Section for Psychosomatic Medicine, Division of Mental Health and Dependency, University Hospital Oslo, Oslo, Norway; ^6^Department of Health Psychology, University of Leuven, Leuven, Belgium; ^7^Division of Internal Medicine, Department of Psychosomatic Medicine, University and University Hospital Basel, Basel, Switzerland; ^8^Department of General Internal Medicine and Psychosomatics, University of Heidelberg, Heidelberg, Germany; ^9^Department of Medical and Clinical Psychology, Tilburg University, Tilburg, Netherlands; ^10^Department of Medicine and Psychiatry, University of Zaragoza, Zaragoza, Spain; ^11^Instituto de Investigación Sanitaria Aragón (IIS Aragón), CIBERSAM, National Institute of Health Carlos III, Zaragoza, Spain; ^12^University of Oxford, Oxford, United Kingdom; ^13^Department of Psychosomatic Medicine and Psychotherapy, Nuremberg General Hospital, Paracelsus Medical University, Nuremberg, Germany; ^14^Institute for Psychosomatic Medicine and Psychotherapy, University Clinic Hamburg-Eppendorf, Hamburg, Germany

**Keywords:** somatic symptom disorder, bodily distress disorder, functional disorders, research agenda, europe, delphi study, expert survey, EAPM

## Abstract

**Background:** Somatic Symptom Disorders (SSD), Bodily Distress Disorders (BDD) and functional disorders (FD) are associated with high medical and societal costs and pose a substantial challenge to the population and health policy of Europe. To meet this challenge, a specific research agenda is needed as one of the cornerstones of sustainable mental health research and health policy for SSD, BDD, and FD in Europe.

**Aim:** To identify the main challenges and research priorities concerning SSD, BDD, and FD from a European perspective.

**Methods:** Delphi study conducted from July 2016 until October 2017 in 3 rounds with 3 workshop meetings and 3 online surveys, involving 75 experts and 21 European countries. EURONET-SOMA and the European Association of Psychosomatic Medicine (EAPM) hosted the meetings.

**Results:** Eight research priorities were identified: (1) Assessment of diagnostic profiles relevant to course and treatment outcome. (2) Development and evaluation of new, effective interventions. (3) Validation studies on questionnaires or semi-structured interviews that assess chronic medical conditions in this context. (4) Research into patients preferences for diagnosis and treatment. (5) Development of new methodologic designs to identify and explore mediators and moderators of clinical course and treatment outcomes (6). Translational research exploring how psychological and somatic symptoms develop from somatic conditions and biological and behavioral pathogenic factors. (7) Development of new, effective interventions to personalize treatment. (8) Implementation studies of treatment interventions in different settings, such as primary care, occupational care, general hospital and specialty mental health settings. The general public and policymakers will benefit from the development of new, effective, personalized interventions for SSD, BDD, and FD, that will be enhanced by translational research, as well as from the outcomes of research into patient involvement, GP-patient communication, consultation-liaison models and implementation.

**Conclusion:** Funding for this research agenda, targeting these challenges in coordinated research networks such as EURONET-SOMA and EAPM, and systematically allocating resources by policymakers to this critical area in mental and physical well-being is urgently needed to improve efficacy and impact for diagnosis and treatment of SSD, BDD, and FD across Europe.

## Introduction

Persistent distressing physical symptoms present a huge individual and societal burden and unmet clinical need. For example, in the 2016 Global Burden of Disease Study, low back pain (LBP) without any diagnosed underlying medical condition is the leading cause for years lived with disability (YLDs) (1), with an estimated cost of €57.6 million (95% Uncertainty Interval (UI) 40.8–75.9). Its global prevalence rose 18% in 2016 alone, and it is the leading cause of YLDs in Europe (1). Hence, from a European perspective, the symptoms most contributing to YLDs are in the realm of persistent distressing physical symptoms, or so-called Medically Unexplained Symptoms if they occur without a diagnosed medical condition (2–4). Persistent distressing physical symptoms are associated with mental disorders, especially major depression(5–12), which is related to somatization, that is the expression of psychological distress into somatic symptoms (13, 14). Major depression is also amongst the top five of the 2016 global burden of disease study (1). This huge burden calls for a sustainable European mental health research agenda and health policy in this field.

In the last decade, the classification of persistent distressing symptoms and its research domain has been in significant transition and this is related to different views about conceptualization (15). It has been argued that somatization is an underlying concept that expresses views on the concept of disease. They may be influenced by new knowledge about pathogenesis but also by new angles to approach the subject of combined somatic and psychological problems or disorders (16). The ongoing debates about classification can be seen as an example of this (15). The former DSM-IV somatoform disorder section mostly focused on the criterion that physical symptoms should medically unexplained (17), which left diagnostic and treatment difficulties unresolved (18–20). This changed with the introduction of the DSM-5 Somatic Symptom Disorders (SSD) in 2013. In DSM-5 the nature of the physical symptoms, i.e., being medically unexplained or not, is no longer a criterion. Instead, DSM-5 focuses on the way a patient emotionally, cognitively and behaviorally copes with the physical symptoms (21). According to the SSD classification, patients suffering from chronic medical conditions can also be diagnosed and receive treatment. This in its own right poses new diagnostic and treatment challenges (22, 23).

The proposed ICD-11 beta draft classification of Bodily Distress Disorders (BDD) (24, 25) may differ from SSD (19). This has led to controversy and a proposal to delete BDD from the ICD-11 beta version (26) as it seems hard to discern from Bodily Distress Syndromes (27) that captures many of functional and somatoform disorders (28) and shows similarities with the ICD-10 classification of somatoform autonomous dysfunction (29). Similarly to functional disorders (FD), BDD mainly focuses on medically unexplained physical symptoms for its classification, rather than on their psychological conundrums (30). In order to cover the multitude of aspects described above, in this study, we will use the combined term of Somatic Symptom Disorders (SSD), Bodily Distress Disorders (BDD) and functional disorders (FD), as was done in an earlier study of the EURONET-SOMA network (31).

SSD, BDD, and FD occur frequently and are associated with high personal suffering (32–36), service use (37–40) and costs (3, 12, 41). They are a burden for patients (1) and their families (42), and because of the high level of complexity at diagnostic, treatment, health services and social level (43), they form a diagnostic and treatment challenge for general practitioners (38, 44–47), occupational physicians (48), medical specialists (2), psychotherapists, psychiatrists and allied health professionals alike (49, 50). Because of high disability, and high medical as well as societal costs, they form a substantial challenge to the population and health policy of the European Union. However, because of the described ambiguities in this research domain, knowledge gaps and a diversity of opinions regarding identification, clinical management and policies needed exist. This hampers the introduction of evidence-based policies. Hence, a research agenda is needed as one of the cornerstones of sustainable mental health research and health policy regarding SSD, BDD, and FD in Europe.

Advances in this area of mental health will also result in benefits and cost reductions in the broader arena of health care and disease prevention. For this reason, the EURONET-SOMA group, in collaboration with the European Association of Psychosomatic Medicine (EAPM), endeavored to establish a targeted research agenda, based on experts opinions, with the purpose to motivate funding agencies for grants for this subject and policymakers to systematically allocate resources to this critical area in mental and physical well-being.

## Objective

The present paper aims to report the results from a Delphi study amongst European experts in the field of SSD, BDD and FD to provide insights into the core consensus-based challenges and research priorities relevant to mental and physical health.

## Methods

### Participants

Data for this study were obtained from experts in the field of SSD, BDD, and FD. Experts in all rounds were members of the EURONET-SOMA group. This group was set up in 2016 by Professor Bernd Löwe as part of a funded initiative to improve exchange between European experts with a focus on diagnosis, treatment, and research in the field of SSD, BDD, and FD. EURONET-SOMA has members from Germany, Scandinavia, the Baltic states, Belgium, the Netherlands and the UK. The members were recruited in a three-step procedure as follows.

1. Potential participants with expertise in the field were identified by personal knowledge of Bernd Löwe and his team as well as by checking reference lists from relevant publications.
2. For each potential participant, the respective *PubMed* research record was checked for significant publications in the field.
3. Participants from the Baltic area were “oversampled” in accordance with the goals of EURONET-SOMA.

The first round of the Delphi study was conducted with EURONET-SOMA members. For the second and third round, the Delphi study was kindly adopted by the European Association of Psychosomatic Medicine (EAPM), on the request of EURONET-SOMA and after approval of the EAPM board members. The survey was extended to all EAPM members and associate members, in order to attain a wide representation all over Europe including experts closely collaborating with European experts. The experts approached in the surveys were the same experts who were invited for the workshops, so there was more or less overlap depending on attendance in the workshops and surveys. This resulted in a group of experts from several backgrounds, both researchers also involved in patient care and researchers not involved in patient care. The researchers performed research over the whole realm of basic science, public health, epidemiology, social sciences and communication sciences as well as patient-related clinical research. The experts involved in patient care were from general practice, public health, general hospital psychosomatic settings and mental health settings and involved both doctors and medical specialists as well as psychotherapists and psychologists.

### Design

The present study follows a Delphi design. The Delphi approach is a structured method for collecting opinions of experts concerning a subject of their expertise (51). The essence of a Delphi procedure is the exploration of expert views on a certain topic and giving the option to the experts to react to the input of the other experts in a number of rounds. Since the invention of the Delphi method in the 1950s (52) a commonly used variation of the Delphi method is the estimate-talk-estimate Delphi method that combines assembling of expert opinions on an anonymous basis during surveys with open exchange during workshops by a facilitator. This Delphi method was followed in this study (53). This Delphi procedure had consensus as final aim, which was achieved stepwise in three rounds involving exploration, prioritization and as a final step attaining consensus by an online survey (54). In the last decade, the estimate-talk-estimate Delphi method has shown its value in mental health-related expert studies. The procedure followed in this study was derived from the method followed in ROAMER, a European project funded by the EU FP7 program, that identified research priorities for a research agenda on mental health in Europe with a similar Delphi procedure (55–58).

From July 2016 until October 2017 this three-round Delphi study was performed by a facilitator group assembled by EURONET-SOMA with Prof. Christina van der Feltz-Cornelis, M.D., as lead (IE, UM, UW, OvdB, and CFC). The next sections will discuss the methods, including the participants, for the three rounds separately.

The Delphi study had the following components.

#### 1st round method

The first round took place in July 2016. It started with an EURONET-SOMA workshop in Hamburg attended by 25 experts. In the workshop, an open-ended brainstorm approach was used to identify important knowledge gaps and research challenges in the field of SSD, BDD, and FD. The workshop resulted in 61 challenges over 8 domains, namely: Classification, Outcomes and assessments, Symptoms, Diagnosis, Treatment, Prevention, Communication, Process and Research Coordination.

This workshop was followed by an online survey in order to prioritize the 61 identified challenges. The survey invitation and explanatory texts were written by IE, CFC, and UFM and approved by OvdB and UW. All EURONET-SOMA experts (*N* = 36) were invited by email for participation. They received an email with an individualized survey link that was unique for each participant and could not be forwarded to anyone else. Non-respondents were sent three reminder emails. Twenty-four experts (response rate = 67%) participated in the survey.

The questionnaires for each round consisted of two parts. The first part concerned general information about the respondent, such as questions on demographics (i.e., age, gender, and country) and work-related questions (i.e., primary affiliation, professional degree, fields of research interests and disorder(s) of expertise). The second part concerned the actual study questions. WEBROPOL 2.0, an online software program for gathering and analyzing data, was used for this survey (59).

#### 1st round online survey questionnaire

The first online survey was sent in October 2016. It presented the 61 challenges derived from the workshop and asked the respondents to rate them on a three-point scale: (1) Low priority, (2) Moderate priority, (3) High priority.

#### 1st round analyses

From the online survey, the percentage of experts who rated the challenge as low, moderate or high was calculated. In addition, the average score per research challenge was calculated by the following formula, in which low priority was scored as “1,” moderate priority was scored as “2,” high priority was scored as “3”:

$$\begin{array}{l} \left(\left(Number\;of\;experts{\left(N\right)}^{*}score1\right)+\left(N^{*}score2\right)+\left(N^{*}score3\right)\right)/N\text{\_}total \end{array}$$

Challenges were classified as priorities when they were selected as “high priority” by more than 40% of experts and had an average score > 2. A score higher than two indicates that the priority level of the research challenge is seen as higher than a moderate level. This resulted in 21 prioritized challenges that are shown in order of priority in Table 1.

**Table 1:** Prioritized research challenges as identified in the first wave.

	**Prioritized research challenges**	***N***	**Low (%)^a^**	**Moderate (%)^b^**	**High (%)^c^**	**Mean^d^**
1	What might be relevant mechanisms in symptom development?	24	0	33.33	66.67	2.67
2	What are the most important treatment outcomes for research on somatic symptom disorders?	24	12.5	20.83	66.67	2.54
3	How do psychological and somatic symptoms develop and timely interact with somatic diseases, biomarkers, gut microbiota, health anxiety, brain function?	24	8.33	33.33	58.33	2.5
4	How can we prevent the development or deterioration of somatoform disorders?	24	12.5	29.17	58.33	2.46
5	What can we learn more about mechanisms of somatization in order to improve therapy?	24	8.33	37.5	54.17	2.46
6	Can we develop tailored interventions based on mechanisms in symptom development?	24	16.67	29.17	54.17	2.38
7	What are important moderators/mediators in research on somatic symptom disorders?	24	12.5	37.5	50	2.38
8	Why do we seem to fail to explain MUS/Somatoform Disorder to so many of our patients? How can we improve that?	24	20.83	29.17	50	2.29
9	How can we develop and evaluate strategies to reduce the duration of untreated illness and allow early recognition and treatment of patients with somatoform disorders, (a) in work environments, (b) in primary care, (c) in medical clinics, and (d) in specialist treatment?	24	20.83	29.17	50	2.29
10	How can GPs feedback symptoms' that are non-diagnosable in medical examination?	24	4.17	50	45.83	2.42
11	How can we communicate and explain diagnoses to patients and doctors?	24	4.17	50	45.83	2.42
12	How can we develop a symptom model to supplement the disease model?	24	12.5	41.67	45.83	2.33
13	Can better results be achieved with tailored treatments?	24	16.67	37.5	45.83	2.29
14	How can we develop and evaluate personalized treatment that is specifically tailored to the patients' needs and their response to treatment?	24	16.67	37.5	45.83	2.29
15	What are core sets of instruments to measure key variables, e.g., outcome measurements, prognosis, profiles as targets for treatment, adherence?	24	0	58.33	41.67	2.42
16	How can we develop a short list to measure treatment outcome and what should be the minimum set of questions?	24	8.33	50	41.67	2.33
17	How can we coordinate our research?	24	8.33	50	41.67	2.33
18	What are differences and similarities between functional and associated non-functional syndromes?	24	8.33	50	41.67	2.33
19	How do we integrate parallel development in different medical areas, e.g., gastroenterology, neurology?	24	12.5	45.83	41.67	2.29
20	Should combined somatic and psychiatric symptoms be classified in one uniform way?	24	20.83	37.5	41.67	2.21
21	Do we need to overcome the mind-body-dualism in the classification of symptoms?	24	20.83	37.5	41.67	2.21

a *Percentage of experts that rated this challenges as a low priority (score 1)*,

b *Percentage of experts who rated this challenges as a moderate priority*,

c *Percentage of expects that rated this challenges as a high priority (score 1)*,

d the mean score was calculated by the following formula: ((N ^*^ score 1) + (N ^*^ score 2) + (N ^*^ score 3)) / N_total. Challenges that were selected by more than 40% of the experts as a high priority and had a mean> 2 (indicating an average priority of more than moderate) were seen as priorities. Note: this table only includes the prioritized challenges and not the 61 identified challenges.

#### Second round method

The second round was built on the results from the first stage (60) and provided the experts with the possibility to reflect on the research challenges prioritized in the first round. In the second EURONET-SOMA workshop in Hamburg in November 2016, the 21 prioritized challenges that resulted from the first online survey were presented, discussed and accepted as such by the experts. Upon the advice of the experts, the question “How can we communicate and explain diagnoses to patients and doctors?” was split into two questions, one for patients and one for doctors. The experts also suggested to include the following question regarding the patients' perspective in the survey: “How can we integrate the patients' view upon what is important in the research agenda for SSD, BDD, and FD?” Hence, 23 challenges were prioritized by the experts during the workshop to include in the next online survey.

As it emanated from the demographic variables that only Northwestern European experts participated in the first round, and the aim was to provide a research agenda for Europe as a whole, the EURONET-SOMA working group decided during this workshop to invite the European Association of Psychosomatic Medicine (EAPM) to adopt the Delphi study and to offer all its members to participate in order to have a wide representation all over Europe.

#### 2nd round online questionnaire

EAPM adopted the study and a second online survey was performed amongst EURONET-SOMA, EAPM experts, and so-called associate EAPM members, that is experts closely collaborating with European experts in the context of EAPM. The EAPM office sent an email to all EAPM members to invite them to participate in the survey. For participating in the survey, the EAPM members had to send an email to the Delphi research group who then invited these EAPM members individually to participate in the survey and sent a link. Non-respondents were sent three reminder emails. In this second round, 33 experts from EURONET-SOMA and EAPM participated.

The second round survey was of an open character and provided the experts the opportunity to give whatever insights they wanted on the way the research challenges should be addressed. The second survey was sent out in December 2016 and presented the 23 identified prioritized challenges and asked the experts with open-ended questions how the identified challenges should be addressed, according to them. This produced actions and research priorities suggested by the experts.

#### 2nd round analyses

The answers provided by the experts were coded independently by two facilitators (UW, IE) into factors representing the same answer, as participants may use different words for the same categories. Double coding was used to ensure that answers were interpreted correctly. These factors were then translated into statements (CFC) in order to define research priorities for every challenge. This list with statements was reviewed by all members of the facilitator group (IE, UW, UFM, OVdB, and CFC) before it entered the third round. At this stage, four main topics were identified for which the expert group would be consulted in the third round workshop.

#### Third round method

The list of challenges generated by the second round was discussed with EURONET-SOMA and EAPM members during a workshop and a session at the EAPM conference in June 28th, 2017 in Barcelona and feedback was requested from the experts concerning several topics that emanated from the survey.

The first topic concerned the domain of classification. The survey showed a wide variety of opinions in this realm, and it was discussed during the workshop if each opinion should be an option for consensus in the following survey. The experts attending the workshop decided that it would be better, for the third round online survey, not to try to establish consensus on the different viewpoints on classification. Related to this, a topic regarding conceptualization of the somatization process, was excluded during the workshop from the following 3rd online survey by the experts as well because of the provided answers, that showed that the experts found this challenge difficult to address in view of the current knowledge gaps and seemed to agree that it was too early for consensus in this field.

Another topic concerned the question regarding what would be the best instruments to assess outcomes, such as disability, functioning, and symptom outcomes, in SSD, BDD, and FD. The answers from the 2nd survey on that question included a range of instruments, items to include in a new instrument and relevant profiles that might be interesting to target. However, as asking consensus on all these questionnaires or items was not deemed feasible in the context of this Delphi study, and as EURONET-SOMA members were already in the process of writing a publication addressing this issue separately (31), the experts decided that it was best to exclude this question from the 3rd round online survey.

Finally, a topic concerned possible moderators that could be relevant for diagnostic profiling of SSD, BDD, and FD. The answers here also included a list of moderators named by the experts; the experts decided that it was best, for the third round online survey, to try to establish consensus on diagnostic profiling as a priority for research, instead of trying to establish consensus on each different possible moderator.

The answers to the open-ended questions were used to elucidate the subject matter in the discussion section.

#### 3rd round online questionnaire

During the first round, in some cases, the complexity of the topics that was experienced and expressed by the experts had induced them to phrase the questions and statements in ways that could be explained in multiple ways. The facilitator group had addressed this in round 2 by exploring the questions as phrased by the experts in the second, open round to provide them the opportunity to give their opinions and suggestions about how these questions should possibly be addressed. The answers to these questions and the suggestions of the experts in the 2nd round workshop were meant to provide the facilitator group with input that enabled them to make choices regarding how to adapt the questions and statements that had been formulated in the first round, for the third round online survey. This way, a list of 16 research priorities was produced for the third and final online survey.

Expert members of the EURONET-SOMA group as well EAPM members were invited for participation. 57 EURONET-SOMA and EAPM experts of a total 164 invited experts (35%) participated. The experts received a public link, filling out the questionnaire was thus anonymous; three reminders were sent. The 3rd online survey was sent August 2017 to EURONET-SOMA and EAPM experts presenting the sixteen research priorities. Experts were asked to rate the importance of the research priorities on a seven-point scale from 1. Totally disagree to 7. Totally agree.

#### 3rd round analyses

Consensus calculation was done by interquartile deviations (IQDs) and median. The IQD presents the distance between the 1st and 3rd quartile. IQD ≤ 1 is considered as consensus on a 7-point scales (61). It means that at least 50% of the respondents have answered within one answer category. As an exploratory analysis, we additionally added the level of consensus of the 40% level; looking at the 30 and 70 percentiles instead of the first and third quartile. This enables us to discern strong consensus (≥50%) from moderate expert consensus (≥40%) in this study.

These answers provided input for the specification of the research agenda. Participants were differentiated into experts engaged both in research and patient care, and expert researchers not providing clinical care, and a sensitivity analysis was performed to explore if consensus differed between those two expert groups. Furthermore, a sensitivity analysis was performed to assess consensus amongst the GPs.

## Results

### Participants

**Figure 1:**
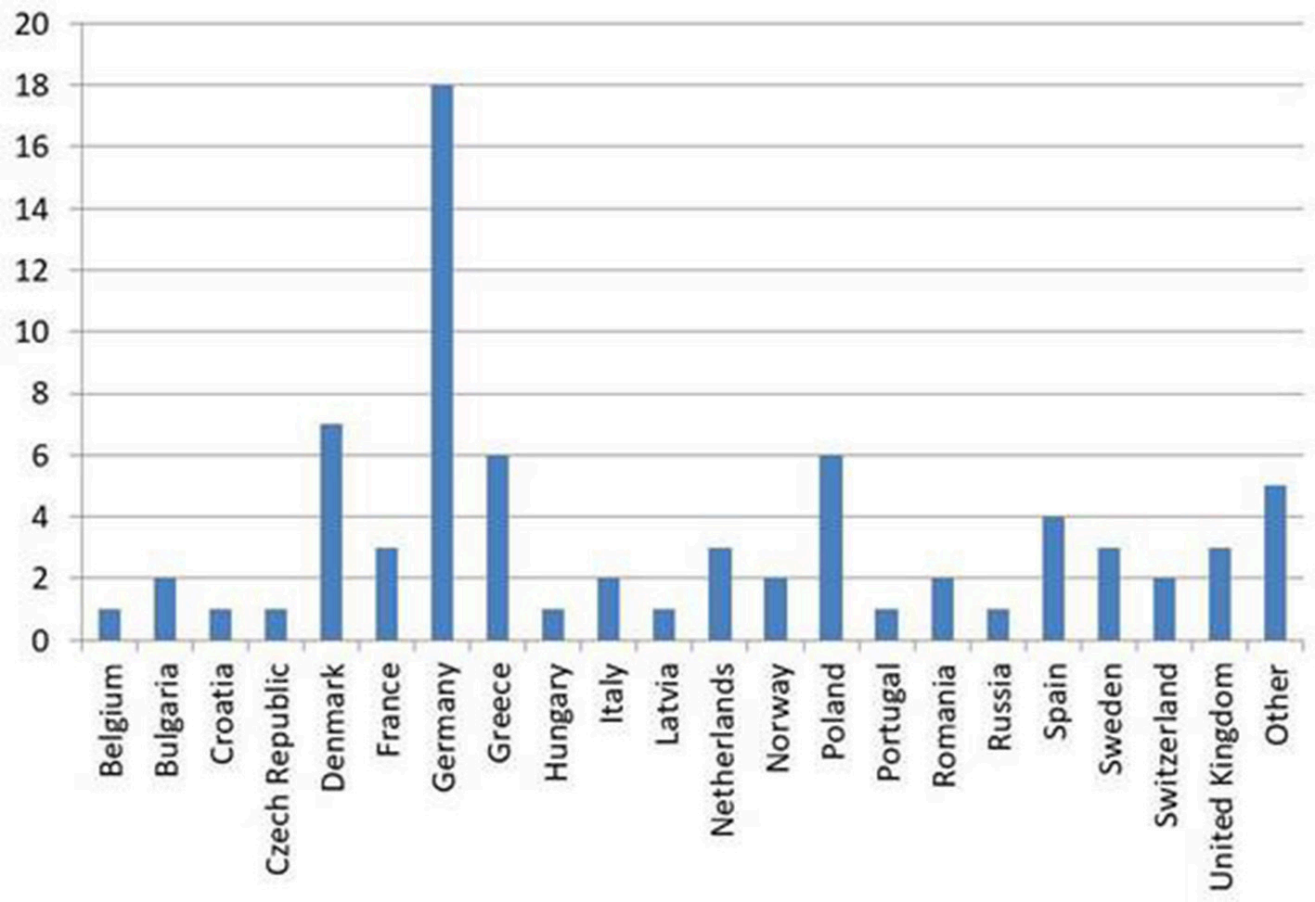
Number of experts per country in the three waves.

In the three rounds with three workshop meetings and three online surveys a total of 75 experts in the field of SSD, BDD and FD participated; 70 from Europe, and 5 associate members of EURONET-SOMA or EAPM who lived in Brazil, Canada, Chile, Israel and New Zealand, and who collaborated closely with European research groups in Denmark, France, Germany, Portugal, and UK. The number of experts per country is shown in Figure 1.

**Figure 2:**
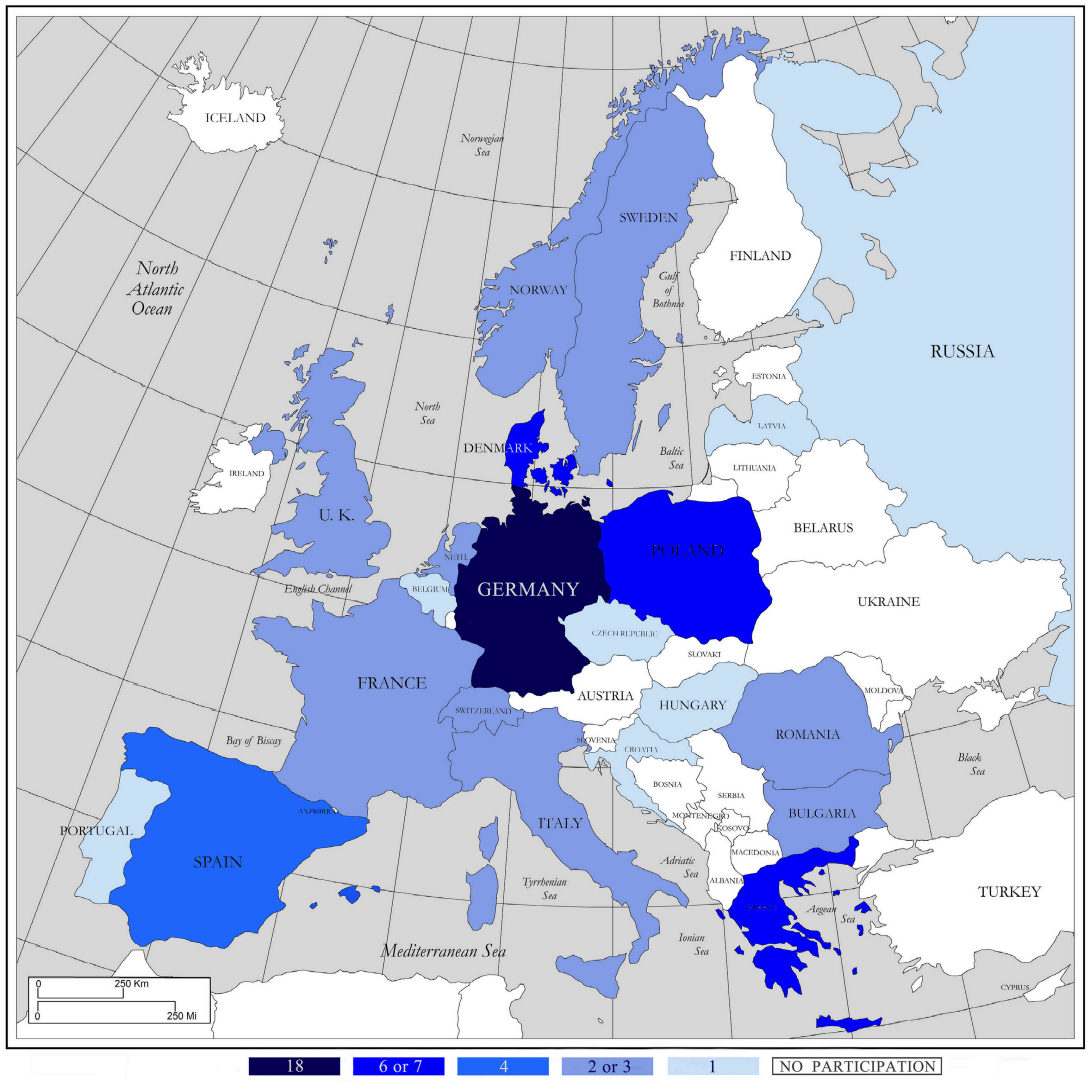
Representation of European countries in the three waves.

The Delphi study involved 21 European countries, with a wide representation of Northern, Western, Eastern and Southern countries, as shown in Figure 2.

Three experts participating in the first survey (4%) were lost to follow up in the two subsequent surveys.

Fifty-seven experts participated in the third survey aimed at consensus. Thirty experts (53%) worked in a university teaching hospital, 19 (33%) in a university, and the others in health care organizations or public health organizations. Twenty-five were a professor, 27 were senior scientist doctors, one had a Master's degree, two were a medical doctor specialized in psychosomatic medicine and 2 held other degrees. The gender distribution (*N* = 56) was balanced with 31 (55%) male and 25 (45%) female. The mean age was 50 years, age range 31–70. Experts came from a variety of research backgrounds including psychological research, clinical trials, health services research, epidemiology/public health and patient-related research as most frequently mentioned. Others were basic science (5), that could be performed by experts only performing research as well as experts also providing patient care, psychosomatic medicine, social sciences, and psychiatry and communication sciences. Forty-nine experts provided more detailed information that could be used to perform the sensitivity analysis. Twenty-two were expert researchers not providing clinical care. Twenty-seven experts were engaged both in research and in patient care. Sixteen (33%) of those were consultant psychiatrists. Six (12%) were engaged in psychosomatic medicine as a specialism in itself, as a medical doctor (2), a psychologist or psychotherapist. Five (10%) were general practitioners.

### Classification

**Figure 3:**
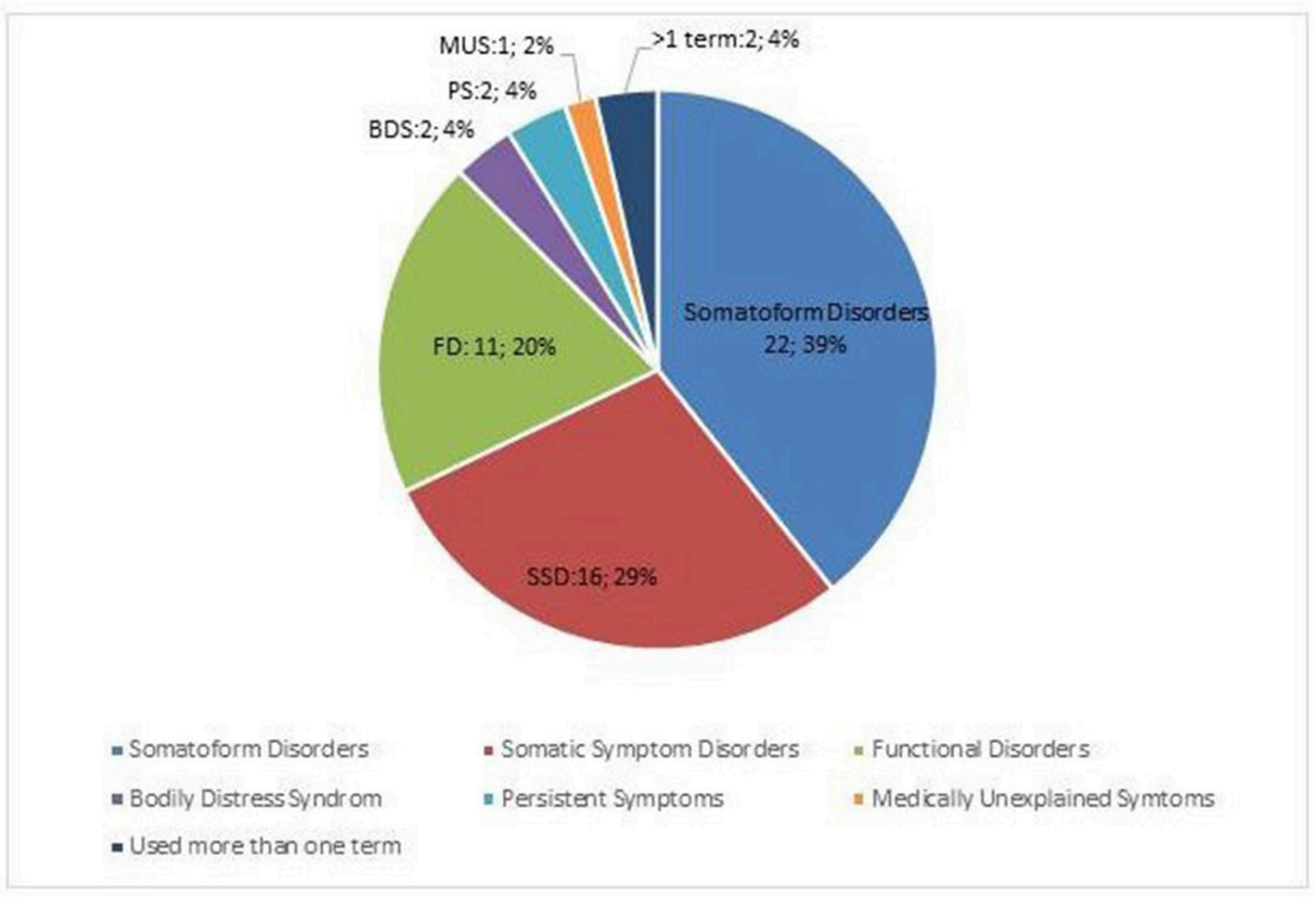
Classifications used by the expert panel.

As an exploratory question, the experts were asked to indicate how they normally classified the disorder(s) to which the survey was directed. As is shown in Figure 3, 22 (39%) indicated to still use the DSM-IV/ICD-10 term somatoform disorders (17); 16 (28%) used the term Somatic Symptom Disorders (62); 11 (19%) used the term functional disorders. Other terms, such as Bodily Distress Syndrome (4%), persistent symptoms (4%), Medically Unexplained Symptoms (2%) were less frequently used and the remaining 4% used more than one of the above.

### Research priorities

The research priorities for which consensus was attained amongst the 57 experts participating in the third round are shown in Table 2.

**Table 2:** Level of consensus regarding the priorities.

		**50%**				**40%**			
	**Statements**	***N***	**Missing**	**Mdn^a^**	**IQD^b^**	***N***	**Missing**	**Mdn^a^**	**IQD^b^**
1	**Research into assessment of diagnostic profiles relevant for course and treatment outcome in Somatic Symptom Disorders and related disorders such as Body Distress Syndrome and Functional Disorders**	56	1	6	**1**				
2	**The development and evaluation of new effective treatment interventions for Somatic Symptom Disorders and related disorders**	57	0	6	**1**				
3	**Validation studies on questionnaires or (semi) structured interviews that assess chronic medical conditions in Somatic Symptom Disorders, Body Distress Disorders and Functional Disorders**	57	0	5	**1**				
4	**Research into patients preferences for diagnosis and treatment of Somatic Symptom Disorders and related disorders**	56	1	6	2	56	1	6	**0.9**
5	**Development of new methodologic designs to identify and explore mediators and moderators of the course and treatment outcome of Somatic Symptom Disorders, Body Distress Disorders and Functional Disorders**	57	0	6	2	57	0	6	**1**
6	**Translational research exploring how psychological and somatic symptoms in SSD, BDD and FD develop from somatic diseases and pathogenic factors as indicated by biomarkers, from cognitive and behavioral factors and from brain function**	56	1	6	1.75	56	1	6	**1**
7	**The development of new, effective interventions to personalize treatment**	57	0	6	1.5	57	0	6	**1**
8	**Conducting implementation studies of treatment interventions in different treatment settings, such as primary care, occupational care, general hospital and specialty mental health settings**	57	0	6	2	57	0	6	**1**
9	Research into effectiveness of communication models with colleagues in Somatic Symptom Disorders, Body Distress Disorders and associated functional disorders	57	0	6	2.5	57	0	6	1.6
10	The development and evaluation of new effective methods for prevention and early recognition of Somatic Symptom Disorders and related disorders	57	0	6	2	57	0	6	2
11	Research into how GPs should provide feedback to the patient on symptoms that are incompatible with medical disease after medical examination	57	0	6	2	57	0	6	2
12	Research into moderators/mediators of the course and treatment outcome of Somatic Symptom Disorders, Body Distress Disorders and associated functional disorders	57	0	6	2	57	0	6	2
13	The development of new and effective communication models between GPs and patients in treatment of Somatic Symptom Disorders and related disorders	56	1	6	2	56	1	6	2
14	The development and evaluation of effective consultation-liaison models between GPs. Medical specialists and psychotherapists and psychiatrists in different health service systems	57	0	6	2	57	0	6	2
15	The formation of sustainable research networks with participants from primary care, mental health care, medical health care systems such as gastroenterology and neurology, for research into Somatic Symptom Disorders	57	0	6	2	57	0	6	2
16	The exploration of research needs of patient associations in the field of Somatic Symptom Disorders, Body Distress Disorders and associated functional disorders	57	0	5	2	57	0	5	2

a *Median score*,

b *IQD, Interquartile Deviation, IQD ≤ 1 indicates group consensus. We only looked whether there was consensus by the 30-70th percentile (thus 40%) when there was no consensus on the 50% level. Research priorities with consensus are in bold*.

Strong expert consensus, which is 50% or above, existed with regard to the following research priorities:

Research into the assessment of diagnostic profiles relevant to course and treatment outcome of SSD, BDD, and FD.

Development and evaluation of new, effective treatment interventions for SSD, BDD, and FD.

Validation studies for questionnaires or semi-structured interviews assessing chronic medical conditions in the context of SSD, BDD, and FD. The experts indicated that with the changing domain and the explicit inclusion of chronic medical conditions in SSD in the DSM-5, the current lack of valid questionnaires or semi-structured interviews is a major gap that has to be addressed in research.

A moderate expert consensus of 40% existed on the following research priorities:

Research into patients' preferences for diagnosis and treatment of SSD, BDD, and FD.

Development of new methodological designs to identify and explore mediators and moderators of the course and treatment outcome of SSD, BDD, and FD.

Translational research exploring how somatic and psychological symptoms develop in *multifactorial etiopathogenesis* as indicated by biomarkers, cognitive and behavioral factors and brain function, for SSD, BDD, and FD.

The development of new effective interventions to personalize treatment, for SSD, BDD, and FD.

Conducting implementation studies of treatment interventions for SSD, BDD, and FD in different treatment settings, such as primary care, occupational care, general hospital and specialty mental health settings.

Expert consensus was not attained in fields such as conceptual models, classification, and terminology, for which the experts indicated two main reasons. One was divergent views on the inclusion of chronic medical conditions in this domain, as opposed to a focus limited to Medically Unexplained Symptoms. The other reason was that experts felt that the knowledge gap regarding underlying pathogenic mechanisms should be addressed first.

### Sensitivity analysis

The sensitivity analysis explored possible differences in consensus-based research priorities amongst experts who also provided patient care (*N* = 27, 47%), vs. experts who only did research. This analysis was performed in a subset of the 3rd round experts, namely 49 experts of whom more details were available that enabled to perform the sensitivity analysis. The results are shown in Table 3.

**Table 3:** Sensitivity analysis.

	**Patient care group**				**Research only group**			
**Statements**	***N***	**Missing**	**Mdn^a^**	**IQD^b^**	***N***	**Missing**	**Mdn^a^**	**IQD^b^**
**STATEMENTS WITH CONSENSUS IN BOTH GROUPS**								
The development and evaluation of new effective treatment interventions for Somatic Symptom Disorders and related disorders	27	0	7	**1**	22	0	6	**1**
**STATEMENTS WITH CONSENSUS IN RESEARCH PLUS PATIENT CARE GROUP ONLY**								
The development of new, effective interventions to personalize treatment	27	0	7	**1**	22	0	6	2
Research into assessment of diagnostic profiles relevant for course and treatment outcome in Somatic Symptom Disorders and related disorders such as Body Distress Syndrome and Functional Disorders	27	0	6	**1**	21	1	6	1.5
The development and evaluation of effective consultation-liaison models between GPs, medical specialists and psychotherapists and psychiatrists in different health service systems	27	0	6	**1**	22	0	6	2
Research into patients preferences for diagnosis and treatment of Somatic Symptom Disorders and related disorders	27	0	6	**1**	21	1	6	2
Research into effectiveness of communication models with colleagues in Somatic Symptom Disorders, Body Distress Disorders and Functional Disorders	27	0	6	**1**	22	0	5.5	3
**STATEMENTS WITH CONSENSUS IN ‘RESEARCH ONLY’ GROUP**								
Translational research exploring how psychological and somatic symptoms in SSD, BDD and FD develop from somatic diseases and pathogenic factors as indicated by biomarkers, from cognitive and behavioral factors and from brain function	27	0	6	2	21	1	7	**1**
**CONSENSUS IN NONE OF THE GROUPS**								
The formation of sustainable research networks with participants from primary care, mental health care, medical health care systems such as gastroenterology and neurology, for research into Somatic Symptom Disorders	27	0	6	2	22	0	6.5	2
Research into moderators/mediators of the course and treatment outcome of Somatic Symptom Disorders, Body Distress Disorders and Functional Disorders	27	0	6	2	22	0	6	2
Development of new methodologic designs to identify and explore mediators and moderators of the course and treatment outcome of Somatic Symptom Disorders, Body Distress Disorders and Functional Disorders	27	0	6	2	22	0	6	1.25
The development and evaluation of new effective methods for prevention and early recognition of Somatic Symptom Disorders and related disorders	27	0	6	2	22	0	6	2.25
Research into how GPs should provide feedback to the patient on symptoms that are incompatible with medical disease after medical examination	27	0	6	2	22	0	6	2
Conducting implementation studies of treatment interventions in different treatment settings, such as primary care, occupational care, general hospital and specialty mental health settings	27	0	6	2	22	0	6	3
Validation studies on questionnaires or (semi) structured interviews that assess chronic medical conditions in Somatic Symptom Disorders, Body Distress Disorders and Functional Disorders	27	0	5	2	22	0	6	2
The development of new and effective communication models between GPs and patients in treatment of Somatic Symptom Disorders and related disorders	27	0	6	2	21	1	5	2.5
The exploration of research needs of patient associations in the field of Somatic Symptom Disorders, Body Distress Disorders and Functional Disorders	27	0	5	2	22	0	5	2.25

a *Median score*,

b *IQD, Interquartile Deviation, IQD ≤ 1 indicates group consensus at 50% level*.

The experts who also provided patient care and the experts who were only involved in research shared the following priority of the whole group, namely:

The development and evaluation of new effective treatment interventions for Somatic Symptom Disorders and related disorders

The experts who also provided patient care shared the first priority of the whole group, namely:

Assessment of diagnostic profiles relevant to course and treatment outcome.

They identified the following research priorities that were a 40% consensus priority for the whole group, as 50% consensus priority:

Development of new, effective interventions to personalize treatment.
Research into patients preferences for diagnosis and treatment of Somatic Symptom Disorders and related disorders.

Furthermore, they identified extra research priorities that concerned the development of interventions in the realm of communication, as shown in:

The development and evaluation of effective consultation liaison models between GPs, medical specialists and psychotherapists and psychiatrists in different health services for SSD, BDD, and FD;
Research into the effectiveness of communication models with colleagues in SSD, BDD, and FD.

The experts who were only involved in research further identified the research priority that was a 40% consensus priority for the whole group, as 50% consensus priority:

Translational research exploring how psychological and somatic symptoms in SSD, BDD, and FD develop from somatic diseases and pathogenic factors as indicated by biomarkers, from cognitive and behavioral factors and from brain function.

Furthermore, an analysis was performed amongst the five GPs participating in the third consensus round, in order to explore if they had a consensus about other research priorities than the whole expert group. This was partly the case. Their first research priority was

a. Development of new, effective interventions to personalize treatment.

This is research priority # 7 for the whole group. Their second consensus priority was

b. Research into how GPs should provide feedback to the patient on symptoms that are incompatible with medical disease after medical examination.

This is research priority #11 for which there was no consensus in the whole group. Their third consensus research priority was

c. Development of new and effective communication models between GPs and patients in the treatment of SSD.

This is research priority #13 for which there was no consensus in the whole group.

## Discussion

This Delphi study presents knowledge gaps, challenges and research priorities identified by 70 experts from 21 European countries and 5 experts from other countries collaborating closely with European research groups in the field of SSD, BDD and FD. It is the first Delphi study establishing a European research agenda based on the input from renowned European experts in the field.

### High response rate

The response rate in the first round was high: 67%. It was not possible to establish the precise response rate in the 2nd and third round, as the EURONET-SOMA and EAPM networks overlap and EAPM experts were approached indirectly via their own network. However, taking the overlap into account, an estimate of a total of experts that was approached was 164. Hence the response rate for the 3rd round is deemed to be approximately 35% (57 of 164), which is still a high response rate for Delphi studies.

### Knowledge gap: classification and conceptualization

It is clear from the results that the experts did not all embrace the DSM-5 SSD classification (62). The most used classification is that of DSM-IV Somatoform Disorders (17), which from a DSM point of view is no longer valid. A sizable amount of experts uses the term Bodily Distress Disorder, which is included in the beta version of the ICD-11 (24). However, this term may be abandoned in the final version of ICD-11 (25). The term Medically Unexplained Symptoms is clearly losing ground. During the Delphi procedure, gradually the limited consensus on conceptual models or classification became clear. In the third round workshop, the experts considered it too early to address classification as a research priority. They felt they needed other research first, that would elucidate mechanisms of disease, in order to be able to address classification, terminology and conceptual problems later. They referred to several knowledge gaps as a potential explanation for the lack of consensus. First, and already mentioned, a sizeable amount of experts still uses classifications that focus on the symptoms being medically unexplained, despite the limitations of this conceptualization. Others feel the need to move on, and to incorporate SSD and psychological suffering in the context of chronic medical conditions. Experts raised the need to explore further the role of basic mechanisms such as stress, inflammation, and neuroimmunology. Exploration of neurocognitive and psychological mechanisms in the pathogenesis of these conditions was also flagged up as important (63).

Indeed, the research priorities identified in this Delphi study can provide input to narrow this knowledge gap. Better knowledge of aetiopathogenesis in this field may lead to more efficient patient profiling and personalization of treatments. Ultimately, this will facilitate and improve classification and conceptualization of SSD, BDD, and FD. The eight identified research priorities are summarized below.

### First priority: research into the assessment of diagnostic profiles relevant for course and treatment outcome

Research into the assessment of diagnostic profiles relevant for course and treatment outcome of SSD, BDD, and FD is the first challenge to address. This advice to engage in patient profiling has been made in at least one multidisciplinary guideline (64, 65). Yet, so far, research into the effectiveness of such profiling is lacking. Three challenges lie behind this research priority. First, diagnostic profiling can be defined as a systematic assessment method in the diagnostic phase, that encompasses both biological variables such as biomarkers, psychological variables such as symptomatology, and social factors such as trauma, life events, and social support; then the association of these variables with prognosis and treatment outcome is explored. This should provide researchers and clinicians with profiles of patients that may benefit from a variety of interventions.

Furthermore, this provides researchers with the opportunity to associate biomarkers and other variables with the subjective experience of the patient, a development that is highly needed in view of the so-called “subjectivity gap,” that was first identified as such in a Delphi study aimed at establishing a European research agenda for clinical mental health research (57) in the context of ROAMER (55): the genotyping, imaging or other preclinical studies do not provide input regarding subjective experience. Similarly, they do not relate to diagnostic criteria, and the clinical practice of psychiatry (57). This “subjectivity gap” between basic neuroscience research and clinical reality for patients with mental disorders is considered the main challenge in psychiatric research, for which a shift in research paradigms is required (56).

The diagnostic profiles can be developed from research that explores the role of symptoms or other factors in treatment outcome and identifies the most relevant ones. Experts mention somatic, psychological, personality, social, treatment and resilience as dimensions of factors and state that it would be important to perform research that enables to establish the degree in which each aspect contributes to onset and course of the disorder, which factor would be relevant for treatment purposes and which might predict a variety of outcomes. They underscore that the patient's perception of this would be crucial. This way, possibly subgroups of patients with different mechanisms of symptom pathogenesis can be identified. On this basis, mechanism-based interventions and corresponding differential indications could be developed.

Second, diagnostic assessment is especially important in this patient group, that suffers from what used to be considered medically unexplained symptoms but may now also include medically explained symptoms that pose a serious burden to the patient. Hence, diagnostic profiling may shift from mostly somatically oriented toward more psychologically oriented, as the psychological reaction to the symptoms becomes of utmost importance, irrespective of the cause of the physical symptom.

Third, the disorders addressed in this survey are known to have a wide variety of severity, course, and treatment response; assessing patient profiles that may be relevant for treatment or course will thus form a priority.

Fourth, this priority is also associated with research on endophenotypes and the NIMH's Research Domain Criteria (RDoC) initiative (66, 67).

### Second priority: development and evaluation of new, effective treatment interventions for SSD, BDD, and FD

The second research priority is development and evaluation of new, effective treatment interventions for SSD, BDD, and FD. This is a logical next step after the first priority that concerns aspects of diagnostic assessment, namely profiling and taking chronic medical conditions into account. Several guidelines for treatment of somatoform disorders (65, 68) recommend Cognitive Behavioural Therapy (CBT) in combination with case management and treatment of comorbid anxiety and depression. Existing systematic reviews including two Cochrane reviews report similar recommendations (69–72). However, the changing insights in pathogenesis and ideas concerning conceptualization (73, 74) and the inclusion of chronic medical conditions call for adapted and new interventions. Experts indicate that, in general, treatment should be focused on coping with the symptoms and improving daily activities and quality of life as well as empowerment. However, they also state that providing treatment that diminishes physical symptoms is needed. A Cochrane review claimed the effect of medication to be limited (70). However, several randomized placebo and psychotherapy controlled studies, partly not included in the Cochrane review, have found psychotropic drugs to be effective (75–77). A recent Danish Randomised Placebo-Controlled Trial indicated the effectiveness of Imipramine in functional somatic syndromes (78). This shows us that medication trials are still relevant in this research domain.

### Third priority: validation studies on questionnaires or semi-structured interviews assessing chronic medical conditions in the context of SSD, BDD, and FD

From the sensitivity analysis, several specific priorities in intervention research emanate as follows. The experts indicate as a major challenge in the field of validated questionnaires or interviews to establish if a patient suffers from chronic medical conditions in the context of SSD, BDD, and FD and that the lack of such instruments leads to underreporting. It is a challenge that has to be addressed to enable appropriate interpretation of research findings in this field. The panelists indicated that valid assessment methods are needed in different research designs, such as population or clinical epidemiological studies, clinical trials or proof of concept studies. Although several checklists exist, they have limitations. For example, the Charlson Index (79), only lists chronic medical conditions that have a bad prognosis or high mortality rate. The ICD-10 codes can be noted by use of a classification browser (29). However, clinician-rated checklists cannot be used in population-based epidemiological studies. Hence there may be a need for a validated self-rating scale. We know from many epidemiology studies that self-report by people has moderate to good accuracy compared to records (80–82). Self-report has been found to be valid if the person has to indicate that the person visited a doctor for, and/or did receive treatment for the chronic medical condition from a doctor (37, 83, 84). The Dutch CBS list is a self-rating scale (85) and contains chronic medical conditions that are highly prevalent, such as cardiovascular disease and diabetes mellitus. The scale also assesses the so-called holy seven of the classic psychosomatic domain (86) such as peptic ulcer, bronchial asthma, rheumatoid arthritis, ulcerative colitis, essential hypertension, neurodermatitis, and hyperthyreosis. However, this scale has as the limitation that it is not possible to discern innocent backaches from a hernia, or Irritable Bowel Syndrome from Inflammatory Bowel Disease, without further exploration. Also, we know that patients with SSD, BDD, or FD tend to over-attribute to disease. This is a research challenge that should be addressed.

### Fourth priority: research into patients preferences for diagnosis and treatment of somatic symptom disorders and related disorders

The fourth priority was to perform research into patients preferences for diagnosis and treatment of SDD, BDD, and FD. The research priority concerning exploration of the effectiveness of focusing on patient preferences is in line with developments in the field of shared decision making, which is suggested as an important technique for this. Although this approach has recently been introduced for application in patients with mental disorders in general (87), its application in patients with SSD, BDD, and FD has only been evaluated in a pilot so far. This pilot showed promising results, but Randomized Clinical Trials in this field are urgently needed (88). Furthermore, as patients' needs may change during treatment, stepwise collaborative care models with shared decision making feedback loops using Patient Related Outcome Measurements (89) may be needed. This has been done in depressive disorder (90), but not yet in SSD, BDD, and FD.

### Fifth priority: develop new methodological designs to identify and explore mediators and moderators of course and treatment outcome for SSD, BDD, and FD

The fifth research priority is of a methodological nature, as for these new developments new designs and methods may be needed. One option is to establish a cohort database for patient profiles and nest a Randomized Clinical Trial in such a cohort to evaluate if personalized treatment does indeed improve patient-related outcomes (89). Parameters of interest would include symptom load, attrition rates, patients' active engagement and satisfaction with treatment. Designs for RCTs could incorporate a patient preference arm to explore its effectiveness. Also, they could explore the difference in the effectiveness of interventions for patients identified by screening, and patients referred by general practitioners. This has already been done in depressive disorder (90) and should be explored in SSD, BDD, and FD. Also, studies applying dismantling designs may be conducted to identify key mediators in psychotherapeutic treatments. In such a design, the question is explored if a change in one particular intervention variable in a treatment will change the outcome (91). A dismantling design is a type of therapy outcome study. It allows investigating therapies that have multiple components with the goal of trying to identify those features of the therapy that are either the active mechanisms of change or identify the degree to which specific components add to the magnitude of change attributable to other components (92). One major goal would be to identify which patients could benefit from new interventions, and which do not, as has already been done for anxiety disorders in general practice (93, 94), and should be explored in SSD, BDD and FD as well. Qualitative research can explore what patients actually need. Furthermore, the need for proof of concept studies and studies exploring experimental paradigms are suggested in the context of translational research.

### Sixth priority: translational research exploring (1) how psychological and somatic symptoms develop from somatic diseases and (2) potential pathogenic factors as indicated by biomarkers, cognitive and behavioral factors and brain function, for SSD, BDD, and FD

This priority concerns translational research. This is not only needed for the development of personalized treatment but can also be helpful to fill the conceptual and pathogenic knowledge gap that was indicated as a major gap by the experts. The experts recommend using an inclusive, not an exclusive approach, incorporating both medically unexplained symptoms and bothersome physical symptoms in the context of chronic medical conditions such as in SSD. This should enable researchers to develop a conceptual model and classification that will be closely linked to diagnostic profiling and personalization of treatment for patients. This should not only be based on patient preferences, but also on knowledge of relevant pathogenic mechanisms and prognostic factors. Translational research should explore how somatic and psychological symptoms can develop from somatic diseases, and from stress. The further focus could lie on potential pathogenic factors and possible clinically applicable markers of their predictors. In this context, the experts mention biomarkers, neuro-immunological markers, and inflammation markers, but also cognitive and behavioral factors as well as brain function as assessed by imaging studies. Such factors can play a role in establishing endophenotypes in SSD, BDD and FD (95, 96).

### Seventh priority: development and evaluation of new, effective personalized interventions for SSD, BDD, and FD

This is the seventh priority, that can be seen as a specification of the second priority regarding development and evaluation of new treatment interventions. It is also a research priority emanating from the sensitivity analysis. This shows that this is a generally felt priority, and a high priority from the viewpoint of the researchers also providing patient care. The experts strongly recommend that personalization of treatment should be performed in close collaboration with the patient and while focusing on patient needs. Also, by structured assessment, being able to identify and focus on the core problem that the patient has, or the most impairing problem, and provide specific treatment for that problem. Furthermore, this links with the sixth priority of translational research, as that will be needed in order to enable development of personalized interventions. This approach is supported by several recent studies indicating that somatic symptoms are related to genetic variance across subjects (97–99) As we can see, European experts in the field of SSD, BDD and FD suggest personalized treatment interventions based on translational research and incorporating the patient perspective as priorities.

### Eighth priority: conducting implementation studies of treatment interventions for SSD, BDD, and FD in different treatment settings, such as primary care, occupational care, general hospital and specialty mental health settings

The experts indicate the need to prioritize implementation studies to evaluate which interventions could be best implemented in which health care settings, and in what way. This corroborates the findings that there is a high unmet clinical need and that treatment models may have to be adapted to the respective settings in order to be most effective. Furthermore, the fact that different European countries have different health service models can be taken into account in such studies. Knowledge emanating from such implementation studies may indeed provide knowledge that is pivotal for establishing policies at European level.

### Priorities from the sensitivity analysis: development and evaluation of effective consultation-liaison and communication models for SSD, BDD, and FD.

The experts who not only performed research but also provided patient care identified two extra research priorities, in the field of development of new communication interventions. One was development and evaluation of effective consultation liaison models between GPs, medical specialists and psychotherapists and psychiatrists in different health services for SSD, BDD, and FD. The second was research into the effectiveness of communication models with colleagues in SSD, BDD, and FD. The sensitivity analysis also provided two more consensus research priorities, namely how GPs should provide feedback to the patient on symptoms that are incompatible with medical disease after medical examination, and the development of new and effective communication models between GPs and patients in the treatment of SSD.

Psychiatric consultations to general practitioners have been found to be effective in patients with medically unexplained symptoms and somatoform disorder (100), and more effective for this patient group than psychiatric consultations for depressive disorder in the general practice setting (101). Also, it has been shown that providing a consultation letter to the general practitioner for this patient group is an effective intervention (102). However, the need for research into the effectiveness of communication models is wider than that and also concerns communication between general practitioners and medical specialists other than psychiatrists and psychotherapists. New interventions in this field are needed, to be developed and evaluated in the general practice setting. As patients with SSD, BDD and FS may move in and out of secondary or tertiary care, new interventions in general hospital settings and the specialty mental health settings are equally important (37). In view of the high societal costs, psychiatric consultation models and communication models should also be developed for the occupational health setting. Such models, including blended e-health interventions with decision aids for occupational physicians, have shown to be effective communication models for sick-listed employees with common mental disorders in general (103–105) and to diminish the length of sickness absence. A blended e-health intervention with decision aid was found to be cost-effective from a societal point of reference (106) as well, which makes this even more relevant; however, the effectiveness and cost-effectiveness in SSD, BDD, and FD still have to be established. If researchers develop new interventions that address the communication problems and differing attributions that can exist between patients with SSD, BDD, and FD, and their GPs, medical specialists, psychiatrists, psychotherapists, and allied health professionals, such new interventions may improve attrition, adherence and compliance rates with treatment, and hence lead to more efficient and cost-effective case-management.

### Patient involvement

The experts made several suggestions for patient involvement as one of the actions needed to address the challenges; such as collaborating with them during actual studies and performing a Delphi study amongst patients to explore their needs and preferences. They also made suggestions to explore the role of the patient better in research, as is described above, by methodologies including patient preferences and patient-related outcomes.

### Strengths and limitations of this study

This is the first study providing a European research agenda for SSD, BDD, and FD based upon a Delphi study of experts in the field.

The major strength of the present study lies in the sample of 75 experts representing 21 European countries from various research areas and types of institutions, with a wide representation over Europe. The number of experts may be considered high compared with most other studies describing expert opinions on research (57, 107). The response rate of the surveys was high, 67% in the first round and 35% in the third round. Only 3 experts (4%) from the first survey were lost to the two follow-up surveys. Such low loss to follow up is, in general, seen as a strength in Delphi surveys (107). This underlines the high generalizability of the study findings.

Surveying a priori challenges using open-ended questions is a further strength. This provided an opportunity to suggest new areas while avoiding contamination with former sections. All data were extracted independently by two members of the facilitator group, and the findings were discussed with experts during the scientific workshops.

Another strength of the Delphi study was that we were able to perform a sensitivity analysis yielding additional research priorities in the realm of treatment suggested by experts not only engaged in research but also involved in patient care, and by GP experts. As they were less represented, this might result in a lower prioritization of research priorities relevant for GPs, however, by the sensitivity analysis, those research priorities became visible again. Also, the sensitivity analysis showed that consensus was shared about key priorities amongst experts of different backgrounds and that experts reached out of their own terrain by supporting or suggesting research priorities relevant for experts in other domains. For example, consensus about the research priority for new interventions was reached amongst all experts. And clinically working experts of all disciplines had a consensus about development and evaluation of effective consultation liaison models between GPs, medical specialists and psychotherapists and psychiatrists in different health services as well as research into the effectiveness of communication models with colleagues in this field.

Nonetheless, there may be some limitations as well. Although the response rate of the first round was high (67%), we have only an approximation of the response rates in the last round. Nevertheless, we estimate that this approximates 35%, which is still high for a Delphi and lowers the risk of a biased result. Experts were widely represented over Europe, involved in research and patient clinical care, and in medical disciplines as well as psychological and research background. The number of general medicine experts was relatively low compared to other experts. This may be a limitation, but it may also represent the amount of general interest amongst those different disciplines in the field of SSD, BDD, and FD. As such this may provide us with a relevant reflection of expert opinions in this domain.

Based upon the feedback of the second workshop with experts, the second stage questionnaire did not include consensus assessments with regard to a number of sub-challenges, namely regarding instruments and moderators, but for the main challenges only. Assessing the consensus for all sub-challenges would have specified which instruments and moderators are perceived as a priority. However, utilizing this approach would have resulted in a quite extensive questionnaire, forming a significant time burden for the experts, and was deemed to be out of the scope of this Delphi study.

Another possible limitation was that the first round questions were suggested by the experts and in some cases referred to high-level complexity in the research domain. This complexity is part of the subject matter and thus should be taken into account as was done in the Delphi study. The facilitator group addressed this by exploring these issues during the second open survey and by discussing the outcomes of the surveys in subsequent workshops, as described in the Methods section. This way elucidation of the statements could be provided and this helped in and led to the rewording of the statements in the subsequent rounds.

One remarkable finding, that can be seen both as a limitation and as a strength is that the experts felt that it is too early to establish consensus regarding conceptual modeling and classification of SDD, BDD, and FD. They recognized the need to look at further experimental and translational designs to find a solution to this challenge. This Delphi procedure thus makes clear that there is no consensus yet regarding conceptualization, terminology, and classification. In view of the current ongoing debates mentioned in the Introduction, this can hardly be a surprise. The benefit of this Delphi study is that it points out not only that this is the case, but also why, and what can be done about it. Experts indicate that more knowledge is needed regarding pathogenetic mechanisms and that this will help conceptualization and classification at a later stage. This also is a feeder regarding the priority for translational research and the development of personalized new interventions. This is not unique for the realm of SSD, BDD and FD, as can be seen in the ongoing discussions regarding classification and conceptualization of i.e., schizophrenia, personality disorders and other disorders in the development of the DSM-5 and ICD-11 (62), as well as the development of the RDoC framework as an attempt to integrate views from neuroscience with general psychiatry. This prioritization of translational research in combination with the development of new, and personalized, interventions, points the way to an avenue that can facilitate the development of new classification and conceptual models. This Delphi study shows that this prioritization of research may be relevant for the realm of SSD, BDD, and FD as well (66, 108).

## Conclusion

Based on consensus among renowned European experts in the field of SSD, BDD, and FD, this Delphi study established a research agenda with the following research priorities.

1. Assessment of diagnostic profiles relevant to course and treatment outcome.
2. Development and evaluation of new, effective interventions.
3. Validation studies on questionnaires or semi-structured interviews that assess chronic medical conditions in the context of SSD, BDD, and FD.
4. Research into patients preferences for diagnosis and treatment of Somatic Symptom Disorders and related disorders (SSRD).
5. Development of new methodologic designs to identify and explore mediators and moderators of clinical course and treatment outcomes.
6. Translational research exploring how psychological and somatic symptoms develop from somatic conditions and biological and behavioral pathogenic factors. Such translational research is needed to improve knowledge that may be helpful to develop conceptual models and classification further.
7. Development of new, effective interventions to personalize treatment.
8. Implementation studies of treatment interventions in different settings, such as primary care, occupational care, general hospital and specialty mental health settings. Such research should explore how interventions can best be implemented in the various health care settings and health services systems all over Europe.

Based on the sensitivity analysis, specific research priorities are suggested in the domain of interventions: development and evaluation of new communication and consultation models between professionals, new interventions aimed at shared decision making with the patients, new explanation models for GPs with patients about the nature of their symptoms, as well as communication by GPs with their patients about their symptoms.

As SSD, BDD, and FD are associated with high medical costs and productivity losses, they form a substantial challenge to the population and health policy of Europe. The experts stress the importance of creating funding and coordinated networking as essential action needed in order to target the eight research priorities. Addressing these research priorities will result in increased efficacy and impact for treatment of SSD, BDD, and FD across Europe. There is a high probability of success, given that in Europe skilled academics involved in patient care have organized themselves in several, interrelated research networks such as the EAPM and EURONET-SOMA.

Furthermore, systematic allocation of resources by policymakers to this critical area in mental and physical well-being is urgently needed to improve efficacy and impact for diagnosis and treatment of SSD, BDD, and FD across Europe.

## Author contributions

The Delphi study was led by CvdF-C. CvdF-C, UM, IE designed the first stage questionnaire. BL hosted and led the workshops. IE sent the invitations for the first survey. CvdF-C, UW, IE, UM, and OV built the second stage questionnaire. OV commented on this version. BL, CvdF-C, and WS asked the board of the EAPM to adopt the Delphi study. The Board approved. IE and the EAPM office sent the invitations for the second and third survey. UW, IE, and CvdF-C built the questionnaire for the third and final survey. IE conducted the final stage analyses together with CvdF-C and provided the method section for the manuscript with input from CvdF-C regarding the workshop methodology and questionnaire development. CvdF-C, UW, UM, OV, RS, WK, AL, MS, and BL participated in the Delphi surveys. IE, CvdF-C, UW, UM, OV, RS, WK, AL, MS, WS, BL contributed to the interpretation of the data. CvdF-C wrote the manuscript, which all authors critically revised and approved. The Board of the EAPM approved publication on behalf of EAPM.

### Conflict of interest statement

CvdF-C has received royalties for academic writing. In the last 3 years, she received research grants from the Netherlands Organisation for health research and development (ZonMw). MS has received royalties for academic writing. He is in receipt of research grants from the UK National Institute of Health Research (NIHR). UW has received funding for educational activities on behalf of Norrbotten Region (Masterclass Psychiatry Programme, EAPM 2016 Luleå, Sweden): Astra Zeneca, Janssen, Eli Lilly, Novartis, Otsuka/Lundbeck, Servier, Sunovion, and Shire. RS received funding from the Köhler Stiftung under project no. SO112/10209/16, the Stanley Thomas Johnson Stiftung & Gottfried und Julia Bangerter-Rhyner-Stiftung under project no. PC_28/17, and from the Swiss Cancer League (Krebsliga Schweiz) under project no. KLS-4304-08-2017. The remaining authors declare that the research was conducted in the absence of any commercial or financial relationships that could be construed as a potential conflict of interest.
